# Progress in Diseases of the Heart

**Published:** 1902-02-08

**Authors:** 


### Progress in Diseases of the Heart.

(*Continued from page* 309.)

**Auscultation of the Descending Aorta.** Friedmann[6] describes a phenomenon which is possibly of considerable value in the diagnosis of arterio-sclerosis. In healthy persons the heart sounds may be heard in the back, to the left of the vertebral column as far down as the level of the spine of the scapula. In arterio-sclerosis they are heard, with great force and clear accentuation as low down as the level of the angle of the scapula. The cause of this is not certain, probably many factors contribute; thus, with advancing age, the heart sinks lower and more to the left, and the chest walls are more resonant, while the arterial lesions themselves are probably capable of increasing the rapidity and intensity with which the sounds are transmitted.

**Digitalis.**—Porter[7] summarises as follows his conclusions as to the use of digitalis as a heart tonic. Its composition is very complex, and some of its active principles antagonise others; its cumulative action is due to its contracting the arterioles. It is only of use in lesions of the mitral valve, and even then only for a short time. It should be used only when there is low arterial tension, and marked venous engorgement, and as soon as these conditions are overcome, its action should be suspended. As a diuretic, it is only of value when the arterial tension is low, and the danger of its use is that if pushed to its fullest extent it may completely arrest the functional activity of the kidneys.

**The Senile Heart.**—Dehio[8] says that owing to the arterio-sclerosis of old age there is an increased peripheral resistance, one result of which is that though the power of the heart is sufficient for resting states, it cannot adapt itself to the requirements of increased exercise; it has but little reserve strength. The same amount of work which will make a young heart beat more forcibly and more quickly is apt in the senile heart to produce signs of failure, such as dyspnœa and arrhythmia, with but very little quickening. This comparative failure of acceleration during exercise is probably due to the senile heart being largely unable to react to accelerating stimuli. For instance, whilst in the young total paralysis of the cardio-inhibitory vagus fibres by atropin causes an acceleration of from 30 to 50 beats a minute, in persons over 50 it is less marked or absent. The anatomical cardiac changes are secondary to arterio-sclerosis and consist in hypertrophy of the walls with dilatation of the cavities. Subsequently the muscular fibres gradually atrophy and are replaced by fibrous tissue. This change is most marked in the auricles, which may be converted into passively extensile, but no longer actively contractile sacs.

**Intra-thoracic Tumour of the Posterior Mediastinum.**—Steel[9] has recorded a case of the above which presented some characteristic features. The patient, aged 23, was anæmic and emaciated, with orthopnœa and noisy inspiration and expiration. There was no pulsation over the upper part of the chest, whilst this was very marked over the cardiac region, the whole region seeming to be thrust forward with each systole of the heart. No exaggeration of pulsation could be detected in the neck, nor was there any tracheal tugging, or distended veins either in the neck or on the surface of the chest. The left vocal cord was paralysed. On percussion over the upper median region of the chest, an area of dulness extending about three inches across, and semilunar in shape could be detected. Over the whole cardiac area a loud systolic murmur was audible. The patient suffered so much from distress of breathing that he had to be kept continuously under the influence of morphia. At the post-mortem examination, a lympho-sarcoma was found, forming a hard mass in the upper parts of the middle and posterior mediastina, on which the base of the heart appeared to rest. This accounted completely for the remarkable and uniform thrusting forward of the heart *en masse* during systole. The ordinary impulses of the heart are the apex-beat, the diffuse impulse, corresponding to the movements of the right ventricle, usually observed best in the epigastrium, and the weak impulse of the infundibulum of the right ventricle to the left of the sternum in the second intercostal space. In the above described case none of these could be distinguished, but the whole heart beat against the chest wall. During diastole the heart is flaccid, but in systole it is a rigid mass before which the surrounding parts give way. The case is also interesting, in that the new growth implicated the recurrent laryngeal nerve—a sign which is certainly more common in aneurysm than neoplasm.

[6] Wien. Klin. Woch., No. 25, 1901. [7] Therapist, June 15, 1901. [8] St. Petersburg Med. Woch., No. 9, 1901. [9] Med. Chron., April 1901.

